# Supplementary figures and images for: Prognostic Gene Expression-Based Signature in Clear-Cell Renal Cell Carcinoma
Source: Cancers (Basel). 2022 Aug 1;14(15):3754. doi: 10.3390/cancers14153754 (PMC9367562; doi:10.3390/cancers14153754)

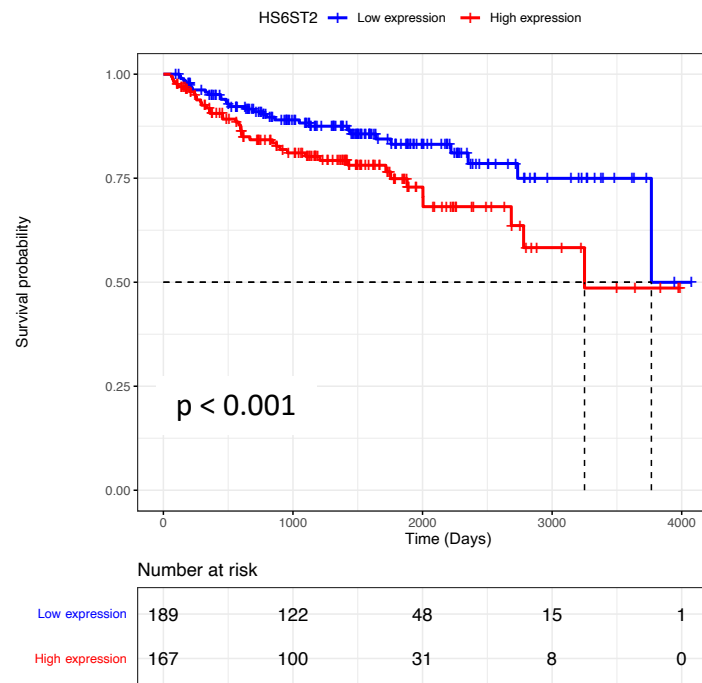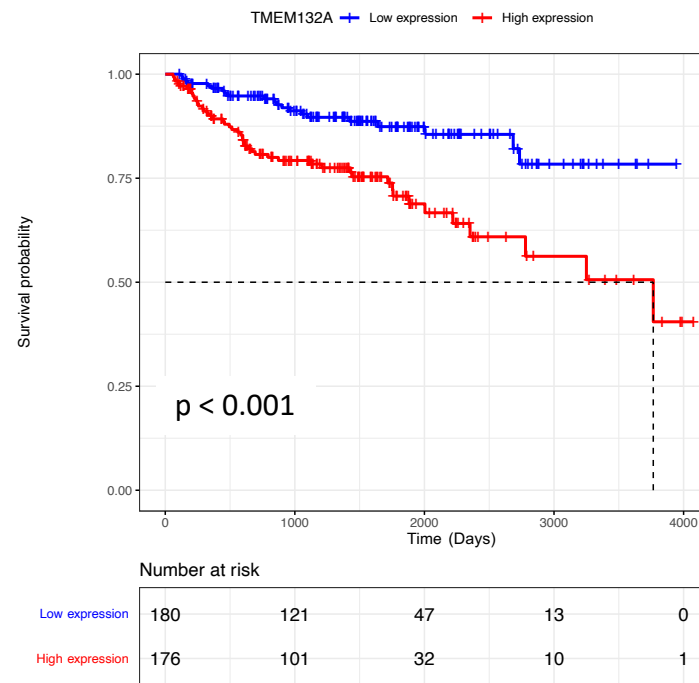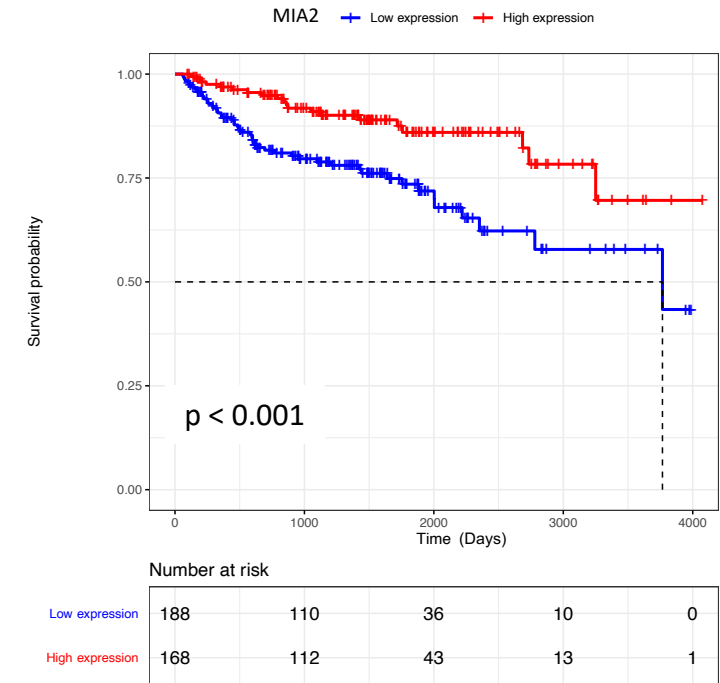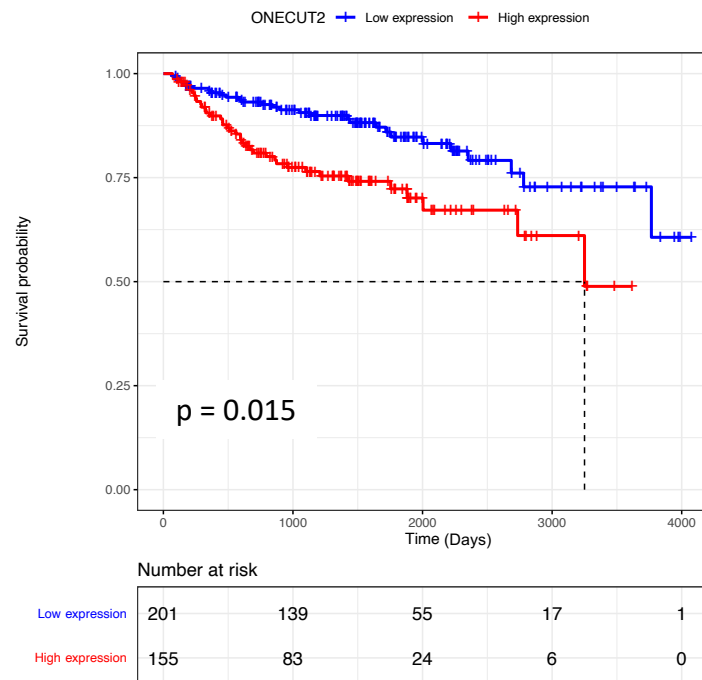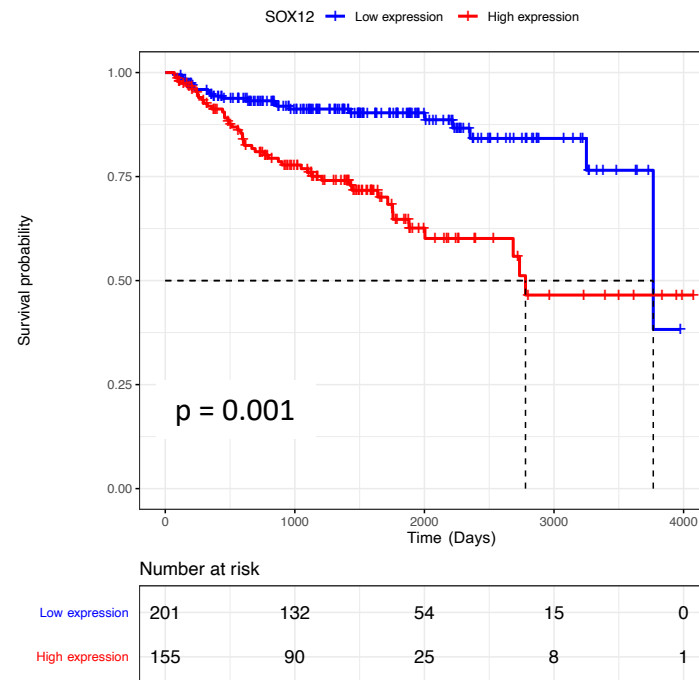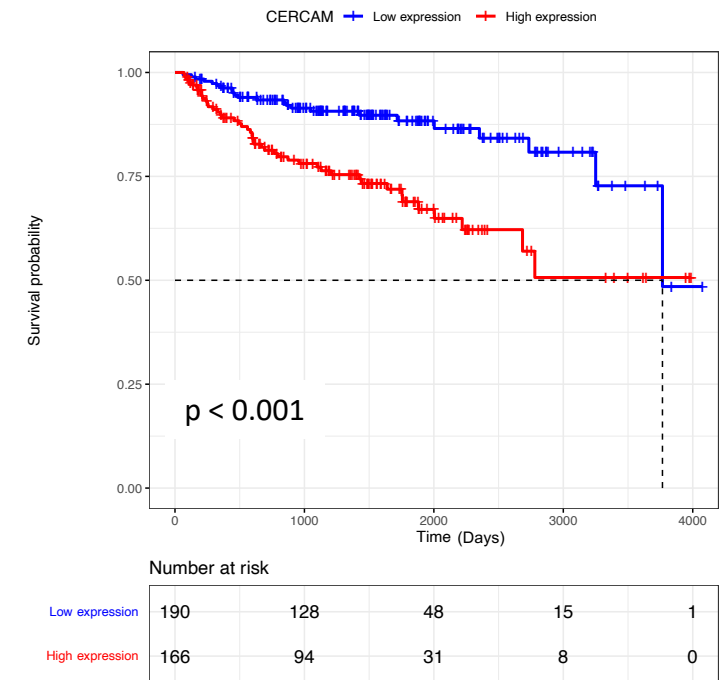

Supplement: Supplementary file 1 [file cancers-14-03754-s001.zip › Supplementary files/ROLDAN ET AL 2022 FIGURE S2 NEW.pdf]

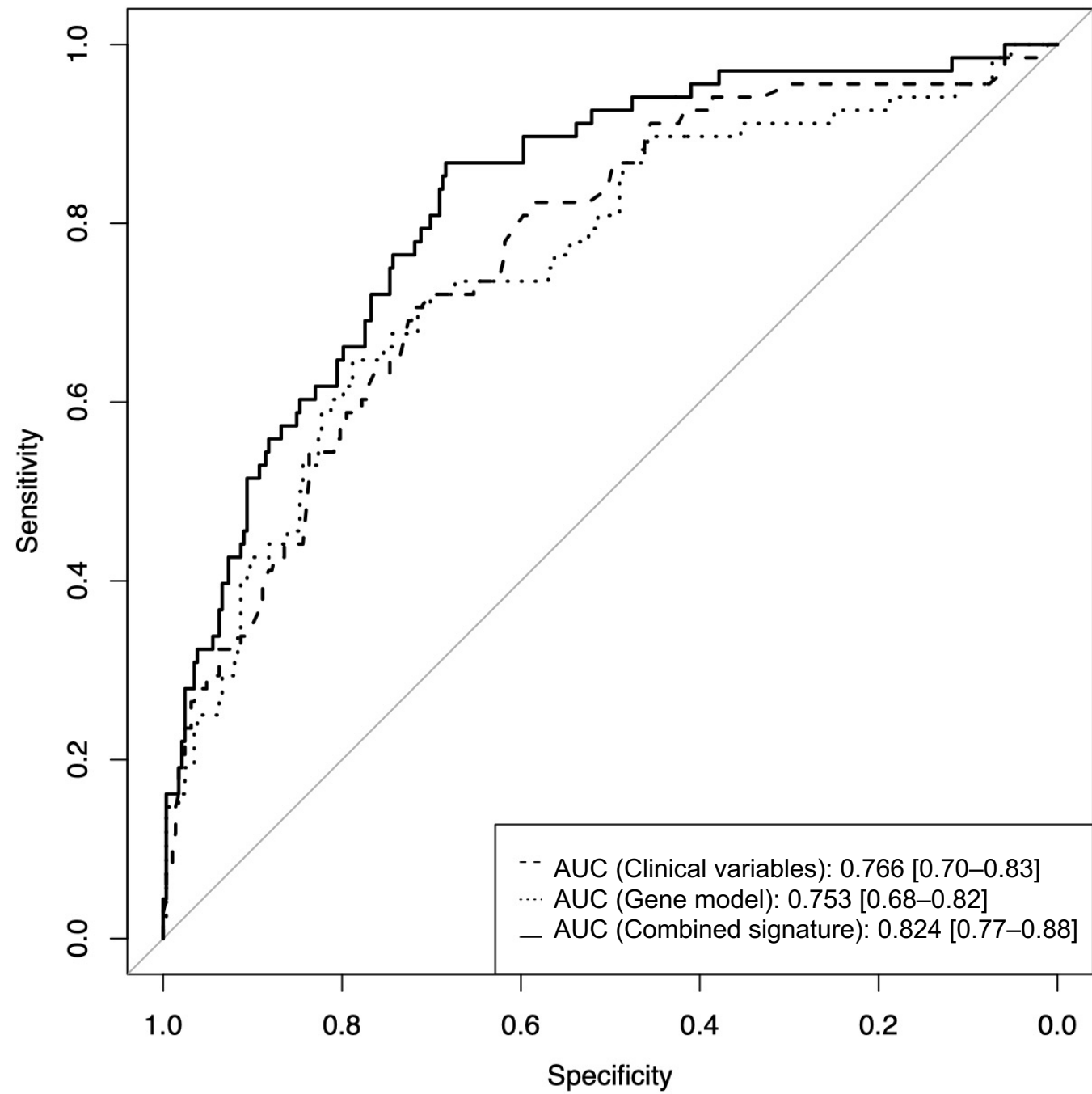

Supplement: Supplementary file 1 [file cancers-14-03754-s001.zip › Supplementary files/ROLDAN ET AL 2022 FIGURE S3.pdf]

(A) SSIGN risk groups

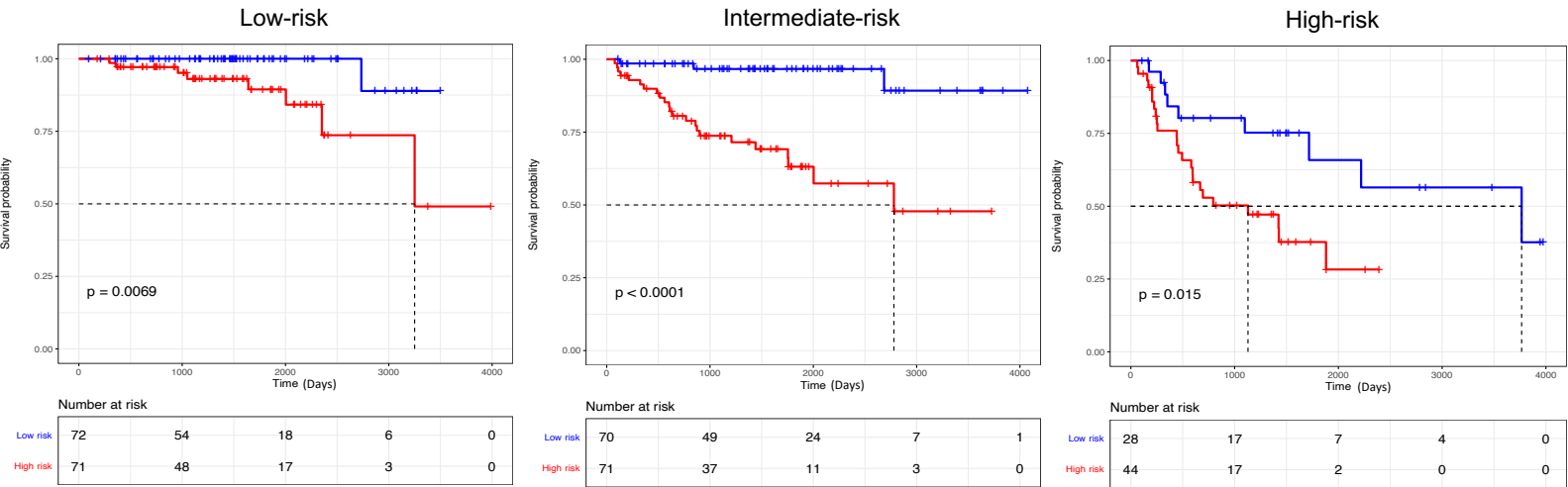

(B) pT stage

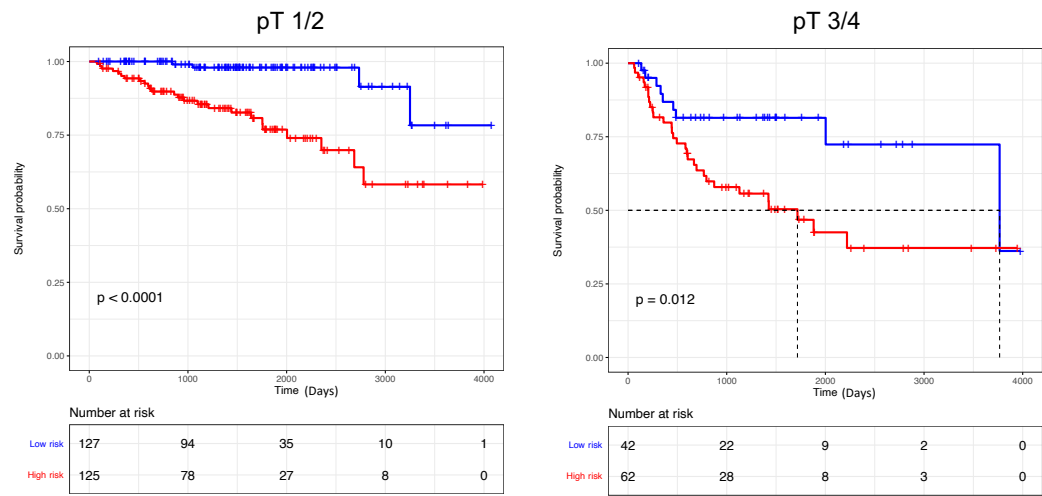

(C) ISUP grade

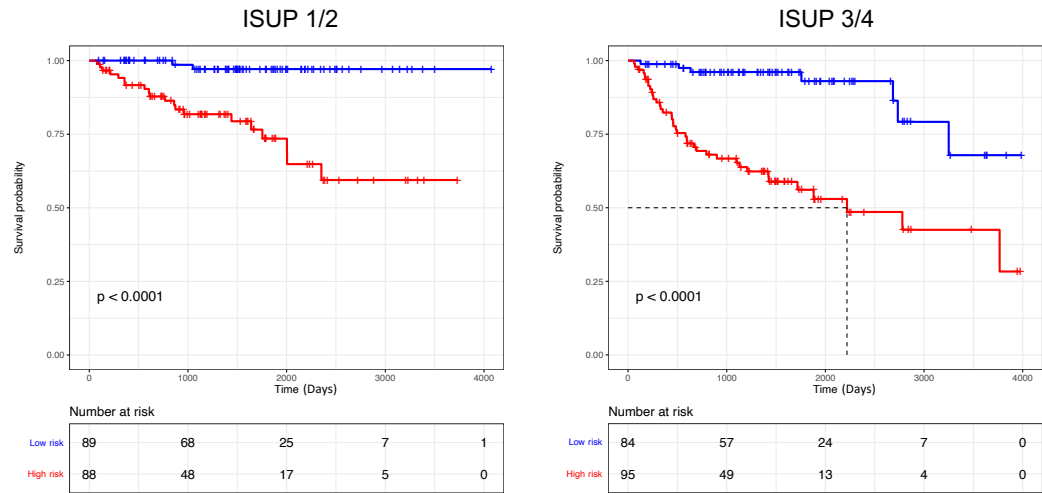

Supplement: Supplementary file 1 [file cancers-14-03754-s001.zip › Supplementary files/ROLDAN ET AL 2022 FIGURE S4_REVISED.pdf]
