# Supplementary material for: Prognostic Gene Expression-Based Signature in Clear-Cell Renal Cell Carcinoma
Source: Cancers (Basel). 2022 Aug 1;14(15):3754. doi: 10.3390/cancers14153754 (PMC9367562; doi:10.3390/cancers14153754)
Supplement: Supplementary file 1 [file ROLDAN ET AL 2022 FIGURE S1.pdf › Supplementary files/ROLDAN ET AL 2022 FIGURE S1.pdf]

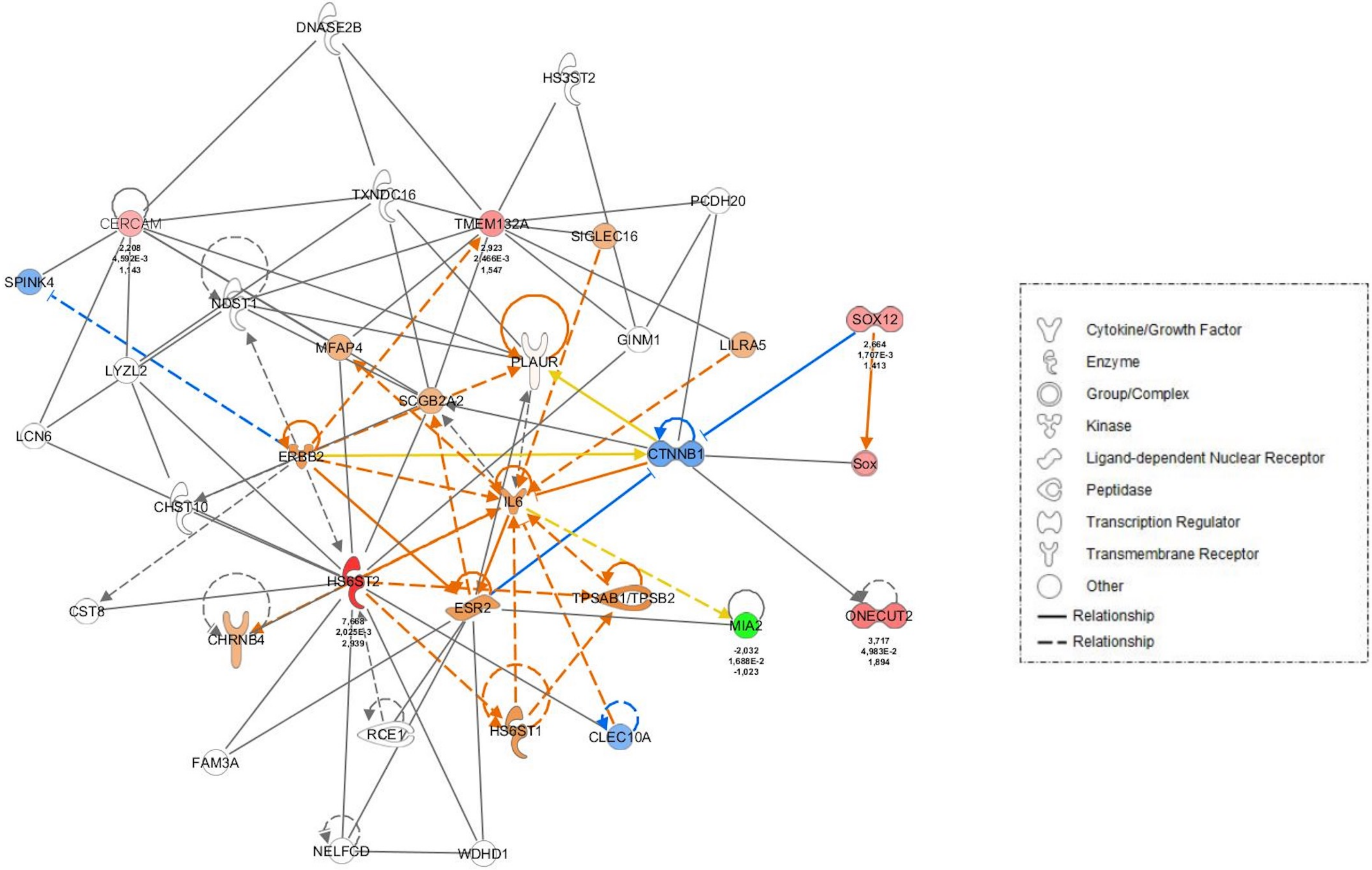

© 2000-2022 QIAGEN. All rights reserved.
